# Single nucleotide polymorphisms at *miR-146a/196a2* and their primary ovarian insufficiency-related target gene regulation in granulosa cells

**DOI:** 10.1371/journal.pone.0183479

**Published:** 2017-08-25

**Authors:** Sung Hwan Cho, Hui Jeong An, Kyung Ah Kim, Jung Jae Ko, Ji Hyang Kim, Young Ran Kim, Eun Hee Ahn, HyungChul Rah, Woo Sik Lee, Nam Keun Kim

**Affiliations:** 1 Department of Biomedical Science, College of Life Science, CHA University, Seongnam, South Korea; 2 Department of Obstetrics and Gynecology, School of Medicine, CHA University, Seongnam, South Korea; 3 Healthcare Bigdata Linkage Platform Team, Chungbuk National University, Cheongju, Chungbuk, South Korea; 4 Fertility Center of CHA Gangnam Medical Center, School of Medicine, CHA University, Seoul, South Korea; University of Toronto, CANADA

## Abstract

MicroRNAs post-transcriptionally regulate gene expression in animals and plants. The aim of this study was to identify new target genes for microRNA polymorphisms (*miR-146a*C>G and *miR-196a2*T>C) in primary ovarian insufficiency (POI). We cloned and transfected *miR-146a*C>G and *miR-196a2*T>C into human granulosa cells and used microarrays and qPCR-arrays to examine the changes in the messenger RNA expression profile. We show *miR-146a*C>G and *miR-196a2*T>C change the mRNA expression patterns in granulosa cell. In each case, mRNAs were up or down-regulated after treatments with *miR-146a* C or G and *miR-196a2* T or C. We found that *miR-146a* led to a significantly altered regulation of the mRNA levels of *FOXO3*, *FOXL2* and *CCND2* compared to controls. We also found that the polymorphisms of *miR-146a* led to a significantly altered regulation of *CCND2* and *FOXO3*. Our results suggest that *miR-146a*C>G and *miR-196a2*T>C can regulate the levels of many of their target transcripts. In addition, specific target genes of *miR-146a*C>G polymorphisms may be involved in granulosa cell regulation.

## Introduction

Primary ovarian insufficiency (POI), also known as premature ovarian failure (POF), is the loss of ovarian function before age 40 [1]. A commonly cited triad for diagnosis is amenorrhea, hypergonadotropism, and hypoestrogenism. If it has a genetic cause, it may be called gonadal dysgenesis [2]. More than 90% of POI cases have unknown causes [3,4].

Recent studies indicate that microRNAs (miRNAs) are involved in ovarian pathologies, such as POI and polycystic ovarian syndrome (PCOS) [5,6]. miRNAs are small, noncoding, single-stranded RNA molecules bind a target messenger RNA (mRNA) [7]. Previous reports showed that miRNAs bind the 3’ untranslated region (UTR) of target mRNAs and modulate their gene expression through deregulation or translational repression [8]. A single miRNA may regulate multiple targets and thus acts as a master controller of gene expression [7,8]. Single nucleotide polymorphisms (SNPs) or mutations occurring in the miRNA gene region may affect the property of miRNAs by altering their expression and/or maturation [9]. miRNAs are also involved in the entire process of ovarian follicle development, including follicle growth and ovulation [10,11]. *miR-146a* is enriched in the oocyte. During bovine oocyte maturation and preimplantation embryo development, *miR-146a* undergoes changes in expression level [12]. The FAS gene, an inducer of oocyte apoptosis during folliculogenesis is an *miR-146a* target [13–15]. *miR-196a2*, which is expressed during oocyte maturation and early bovine embryonic development, regulates the expression of the newborn ovary homeobox (NOBOX) gene during bovine early embryogenesis [16]. NOBOX gene mutations have been reported to cause POI [12,17].

We previously reported the association between Recurrent Spontaneous Abortion (RSA) and POI with polymorphisms of *miR-146a*, *miR-196a2* [12,18]. These results suggest that gene—gene interaction and transcriptional alterations between *miR-146a* and *miR-196a2* may be involved in POI development [12, 19]. Therefore, we hypothesized that transcriptional alterations in *miR-146a*C>G and *miR-196a2*T>C might influence target gene expression and downstream oocyte apoptosis during folliculogenesis.

To identify new target genes expressed in POI, we used a microarray gene expression analysis and validation for novel targets regulated by *miR-146a*C>G and *miR-196a2*T>C in granulosa cells. We identified putative candidates for the genes regulated by the *miR-146a*C>G polymorphism. Our data provide new insights into the potential targets of *miR-146a* in POI regulation.

## Materials and methods

### Ethics statement

The study protocol was approved by the Institutional Review Board of CHA Bundang Medical Center. All study subjects provided written informed consent to participate in the study. All methods used in this study were carried out in accordance with approved guidelines.

### Study participants

All participants were Korean. They were recruited from the Department of Obstetrics and Gynecology of CHA Bundang Medical Center from March 1999 to February 2010. The study group consisted of 113 women (age range, 21–43 years; mean age ± standard deviation [SD], 31.34 ± 4.97 years) who were diagnosed with POI (cessation of menstruation before 40 years of age and two serum FSH levels >40 IU/L). Patients with a history of pelvic surgery, cancer, radiation exposure, autoimmune disorder, or genetic syndromes were excluded. The control group consisted of 227 women (age range, 23–43 years; mean age ± SD, 32.54 ± 3.88 years) who had regular menstrual cycles and at least one live birth. All participants provided informed consent.

### Hormone analysis

To measure FSH, luteinizing hormone (LH), and estradiol (E2) levels, blood samples were collected on the second or third menstrual cycle day. The serum was separated as described previously [20], and hormone levels were measured using radioimmunoassay (E2; Beckman Coulter, Inc, Fullerton, CA, USA) or enzyme immunoassay (FSH and LH; Siemens, Los Angeles, CA, USA) according to the manufacturer’s instructions.

### Genetic analysis of miRNA sequence polymorphisms

Genomic DNA was extracted from anticoagulated peripheral blood using a G-DEX blood extraction kit (iNtRON Biotechnology, Seongnam, South Korea). Nucleotide changes were determined using polymerase chain reaction—restriction fragment length polymorphism (PCR-RFLP) analysis as previously described [21]. Primer sequences for PCR amplification of each polymorphism were as follows: miR-146aC>G: forward 5’-CAT GGG TTG TGT CAG TGT CAG AGC T-3’ and reverse 5’-TGC CTT CTG TCT CCA GTC TTC CAA-3’ (mismatch sequence is underlined); *miR-196a*2T>C: forward 5’-CCC CTT CCC TTC TCC TCC AGA TA-3’ and reverse 5’-CGA AAA CCG ACT GAT GTA ACT CCG-3’. For *miR-146a*C>G and *196a2*T>C polymorphisms, we digested PCR products with *Sac*I and *Msp*I respectively (New England BioLabs, Ipswich, MA, USA) at 37°C for 16 hours, We confirmed the genotype of the two sites by sequencing 10% of the samples.

### *miR-146a*(C>G) and *miR-196a*2(T>C) expression vector construction

To amplify pre-*miR-146a*C or pre-*miR-146a*G from human genomic DNA previously determined to have the G or C genotype, we performed PCR using two primers 5’-GCC GAT GTG TTA TCC TCA GCT TTG-3’ and 5’-ACG ATG ACA GAG ATA TCC CAG-3’ were used to amplify *pre-miR-146a*-C or *pre-miR-146a*-G from each type of human genomic DNA by PCR. PCR products corresponding to pre-miR and its flanking regions (pre-*miR-146a*C, 322 bp; pre-*miR-146a*G, 322 bp; pre-*miR-196a2*T, 345 bp; and pre-miR-196a2C, 345bp) were amplified and cloned into the pcDNA3.1 expression vector (Invitrogen, Carlsbad, CA, USA). The sequences of the vectors were confirmed by direct sequencing; the only difference was in the SNP.

### Cell transfection

Human granulosa cells (KGN) [22] were plated at 1×10^5^ cells per well in a 6-well plate and transfected 24 h later using JetPRIME transfection reagent (Polyplus, Illkirch, France). Each transfection reaction contained 500 ng of miR-146aG (in pcDNA3.1) or 500 ng of miR-146aC (in pcDNA3.1). For controls, we performed mock transfection with scrambled plasmids (Cat No: 1027271, Qiagen, Valencia, CA, USA) transfection with empty pcDNA 3.1 plasmid (Invitrogen, Carlsbad, CA, USA) and used untransfected cells. Total RNA was extracted 24 h after transfection and used for real-time qRT-PCR.

### RNA isolation and cDNA synthesis

Total RNA was isolated using TRIzol^®^ Reagent (Catalog No. 15596–026) and a miRNeasy mini kit (Qiagen) with DNase treatment. cDNA was synthesized by using the miScript II RT Kit (Qiagen). Manufacturers’ protocols were used for all kits. Isolated miRNA was quantified using a Nanodrop ND 1000 (Thermo Fisher Scientific, Waltham, MA, USA), and 500 ng was used for the subsequent cDNA preparation protocol.

### Microarray analysis

The hybridized Human Genome U133A 2.0 Array (Affymetrix, Santa Clara, CA, USA) was scanned and analyzed with the Affymetrix Microarray Analysis Suite version 5.0. The average density of hybridization signals from three independent samples was used for data analysis, and genes with signal density less than 300 pixels were omitted from the analysis. P-values were calculated with two-sided t-tests with unequal variance assumptions. To correct for multiple hypothesis testing, the false discovery rate was calculated.

Differentially expressed genes were selected using both a false discovery rate of less than 0.01 and a fold change greater than 1.5 or less than 21.5. A tree cluster was generated by hierarchical cluster analysis to classify the miRNA-transfected cells. For this analysis, we used average linkage metrics and centered Pearson correlations (Cluster 3.0). Java Tree view 1.1 (http://sourceforge.net/projects/jtreeview/) was used for tree visualization.

### qPCR array analysis

The ExProfile^™^ Gene qPCR Array 96-well-qPCR plate (CS-PAG-062515J1-96B6, GeneCopoeia Inc, Rockville, MD, USA) was used for this study. Each array has a panel of validated, optimized qPCR primers for 32 mRNAs associated with POI as well as the housekeeping genes GAPDH and ACTB, which are used as references to normalize expression. Each well contains a forward primer for the mRNA sequence cross-linked to the 96-well plate. The qPCR Primer Array was performed using 20 μl reaction volumes per well containing 1 μl reverse transcription product and detected using SYBR green, according to the manufacturer’s instructions. The arrays were performed on a Bio-Rad iCycler iQ^™^ instrument (Bio-Rad, Hercules, CA, USA).

### Gene ontology analysis

To analyze the function of those genes, we used the DAVID software (https://david.ncifcrf.gov/)[23], which queries for biological activities according to Gene Ontology (GO) annotations.

### Real-Time RT-PCR detection of *miR-146a* and *miR-196a2*

To evaluate *miR-146a* and *miR-196a2* expression, real-time RT-PCR was used. RNA (500 ng) was used for RT-PCR reactions that were performed using an miScript II RT kit (Qiagen, Hilden, Germany) acording to the manufacturer’s protocol. When reverse transcription reactions are performed, mature miRNAs as well as certain small nucleolar RNAs and small nuclear RNAs are selectively converted into cDNA. Mature miRNAs are polyadenylated by poly(A) polymerase and reverse transcribed into cDNA using oligo-dT primers. The oligo-dT primers have a 3' degenerate anchor and a universal tag sequence on the 5' end, allowing amplification of mature miRNA at the real-time PCR step. Real-time PCR was performed on the Roter-Gene System. RNU6 RNA was used as an endogenous control. All primers were part of SYBR green assays for *miR-146a*, *miR-196a2*, or RNU6 (Qiagen, Hilden, Germany). The cycle number at which the product level exceeded an arbitrarily Ct (Cycle threshold) was determined for each target sequence, and the amount of each miRNA relative to RNU6 RNA was quantified using the formula 2-deltadeltaCt.

### Validation of *miR-146a* target gene expression

Using the TargetRank software (http://genes.mit.edu/targetrank/), the alternative mature forms of *miR-146a*-3p and *miR-196a2*-3p were predicted to have several hundred target genes (using a Target Rankscore ≥0.35, 214 and 166 genes were found for *miR-146a*-3pC and *miR-146a*-3pG, respectively, (supplement information). Because each mature miRNA binds to a distinct set of target genes, different target genes are affected by the miRNAs produced by G or C homozygotes (*miR-146a*-3pC or *miR-146a*-3pG, respectively). We chose POI-related candidate target genes (*FOXO3*, *FOXL2*, *DIAPH2*, *BDNF*, *CCND2*, *and FOXE1*); however, there was no distinct set of POI-related target genes produced by *miR-196a2*-3p T or C homozygotes. To validate the regulation of *miR*-*146a* and *miR*-*196a2* target gene expression, we used real-time qRT-PCR. Total RNA (500 ng) was used for RT reactions that were performed using the miScript II RT kit (Qiagen, Hilden, Germany) according to the manufacturer’s protocol. Real-time PCR was performed on the Roter-Gene system. For the quantitative analysis of predicted target genes, the primers were designed using Primer Premier 3 software (Table 1). GAPDH RNA was used as an endogenous control. All primers were part of SYBR green assays for *miR*-*146a* or *miR*-*196a2* target genes or GAPDH (Bioneer, Daejeon, Korea). The cycle number at which the product level exceeded an arbitrarily Ct (Cycle threshold) was determined for each target sequence, and the amount of each target gene relative to GAPDH RNA was described using the formula2-deltadeltaCt.

**Table 1:** Sequence of primers used for validation of target genes with RT-qPCR.

miRNA	target sequence of 3'UTR*	Primers	Amplicon(bp)
*miR-146a*	FOXO3	F: TCAGTGAGCCAGACTTGCTT	516
		R: CCTTGTCCCTTCCTCAGCTGTTT	
	CCND2	F: ATTGAACCATTTGGGATGGA	325
		R: AAGGGAACAAAATGCCACAC	
	DIAPH2	F: AGGTGCAGCATTCAGAGA	243
		R: AAGTCATGTTGTACCATCACCC	
	BBS9	F: TGCATAGAAAGAGGGGTTGG	242
		R: AACTGGCAAAGGCATATTTTT	
	FOXE1	F: CCCCTTTCCCTTGAGAAATC	309
		R: CCCATTTGGACTGAACCAAG	
	FOXL2	F: TCTTGGCCTTCTCTCACAGG	230
		R: TGCCGGGTTTCACATTTCTC	
	Gapdh	F: AGGTCGGAGTCAACGGATTT	325
		R: ATCTCGCTCCTGGAAGATGG	

* *miR-146a* target prediction: Target Rank (http://genes.mit.edu/targetrank/)

### 3’-UTR reporter gene assay

To generate 3’-UTR luciferase reporter constructs, 3’-UTRs from *CCND2*, *FOXL2*, *FOXO3*, and *FOXE1* mRNAs were cloned downstream of the firefly luciferase gene in the pGL4.13-Control Vector (Promega, Madison, WI, USA). For luciferase reporter assays, KGN cells were plated at 1×10^5^cells per well in 6-well dishes and co-transfected with jetPRIME reagent 24h later (Polyplus, Illkirch, France). Each co-transfection reaction contained 200 ng of pcDNA3::*146a*C or 200 ng of pcDNA3::*146a*G plus 200 ng of pGL4.13::3’-UTR of each target gene construct and 200 ng of pGL4.75 plasmid, which served as the normalization control (Promega, Madison, WI, USA). After 24 h, cells were washed and lysed with Passive Lysis Buffer (Promega, Madison, WI, USA), and firefly luciferase activity was measured using the Veritas Microplate Luminometer (Turner Biosystems, Foster City, CA, USA) and normalized to renilla luciferase activity. We ran all samples in triplicate and replicated the experiment three times.

### Western blot analysis

Proteins were separated using 10.0% SDS polyacrylamide gel electrophoresis and transferred onto nitrocellulose membrane (Millipore, Bedford, MA, USA). The membrane was blocked with 5% non-fat dried milk in TBST (20 mM Tris-HCl, 150 mM NaCl, and 0.1% Tween 20, pH 7.5) for 1 h and incubated overnight at 4°C with each primary antibody. After washing with TBST buffer, membranes were incubated for 1 h at room temperature. Protein bands were visualized using an ECL chemiluminescence system (Amersham, Buckinghamshire, UK). Equal loading of samples was verified by Western blotting for GAPDH. Band intensities were quantified using Adobe Photoshop CS5 (Adobe Systems, San Jose, CA).

### Statistical analysis

All experiments were conducted at least three times. Statistical significance between two groups was evaluated using a two-tailed Student's t test. This statistical analysis was performed with GraphPad Prism software 4.0 (GraphPad Software Inc, San Diego, CA, USA). Differences were considered statistically significant at *P* < 0.05. Associations between gene modulations due to the two miRNAs were examined using a two-sided Fisher exact test. Associations between modulations by any two miRNAs was statistically significant if *P* < 0.001. Correlations between each genotype or allele and FSH, LH or E2 levels were assessed using Kruskal-Wallis and Mann-Whitney tests and *P* < 0.05 was considered statistically significant. These statistical analysis were performed using, MedCalc version 12.1.4 (MedCalc Software).

## Results

### Expression of *miR-146a*C>G and *miR-196a2*T>C in granulosa cells

**Fig 1:**
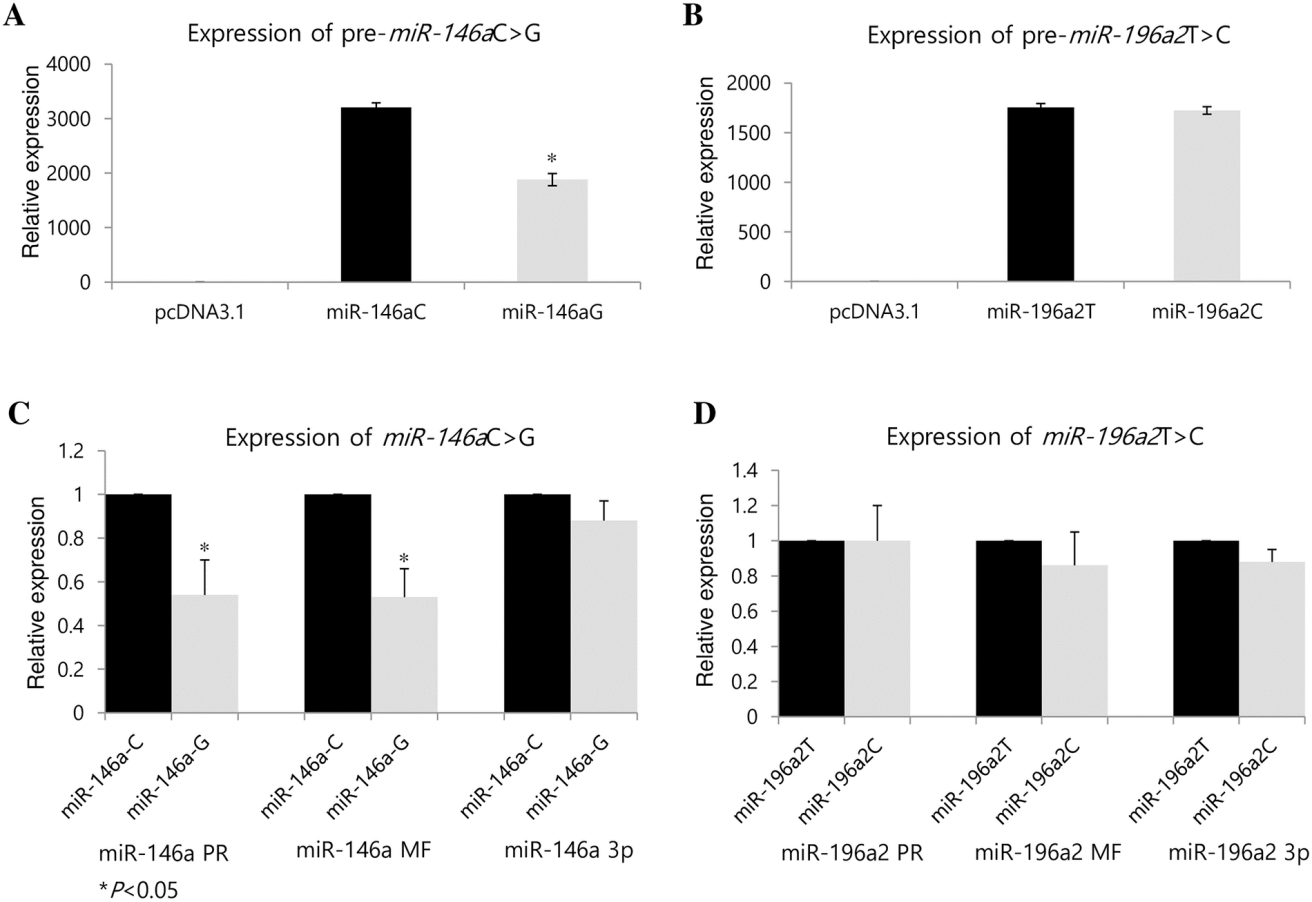
Expression of *miR-146a*C>G and *miR-196a2*T>C. (A) Levels of mature *miR-146a* and (B) Levels of mature *miR-196a2* in cells transfected with *pri-miR-146a*-GG, *pri-miR-146a*-CC or *pri-miR-196a2*TT, *pri-miR-196a2*CC by reverse transcription quantitative polymerase chain reaction. (C) Real-time quantitative PCR was used to detect the expression levels of pre, mature, and mature-3p in *miR-146a*C>G (rs2910164) and (D) *miR-196a2*T>C (rs11614913). Data are reported as means (±SD) from three independent experiments. Data are normalized to the reference RNU6. *P*<0.05 was considered statistically significant. PR: Pre-form, Mature: mature form, -3p: mature form of passenger strand.

*Pre-miR-146a*, *mature-miR-146a*, and *mature-miR-146a*-3p were expressed from C and G alleles; however, the amount of expression differed between the alleles (Fig 1). The mean expression of *pre-miR-146a* in G was 46% lower than in C (*P* = 0.005), expression of *mature-miR-146a* with G was 47% lower than with C (*P* = 0.0004), and *mature-miR-146a*-3p with G was 12% lower than with C (*P* = 0.005) (Fig 1C). The mean pre-miR-196a2 expression with CC was not lower than with TT (*P* = 0.005) (Fig 1D).

### Gene ontology analysis from the microarray data

**Fig 2:**
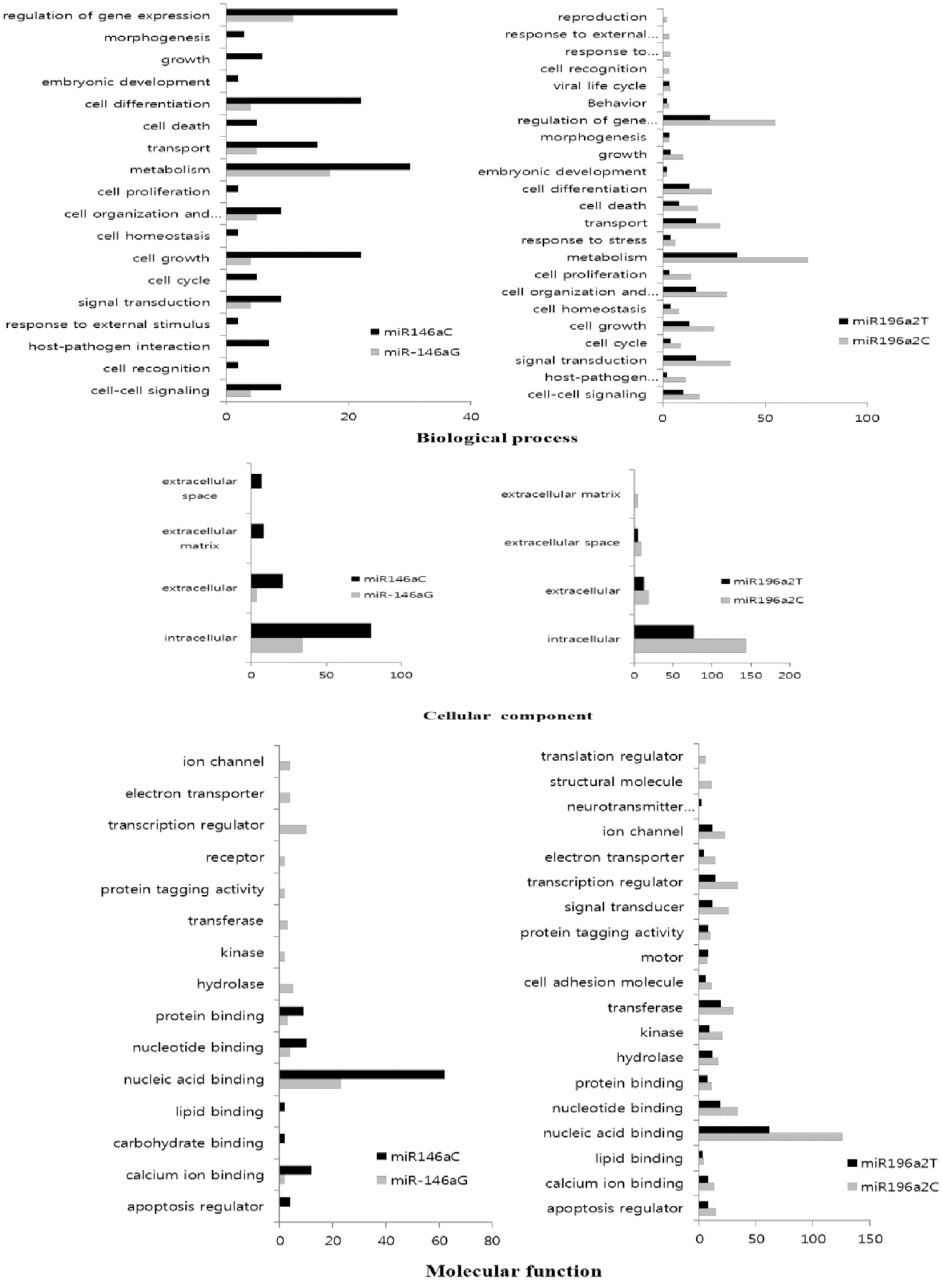
Gene Ontology for 2×up and down-regulated genes by *miR-146a* and *miR-196a2*. The expression of *miR-146a* and *miR-196a2* in granulosa cells was assessed by microarray. (A) Target gene frequency for *miR-146a* [Wild type (C): black, variant (G): silver] and *miR-196a2* [Wild type (T): black, variant (C): silver] in biological process, (B) cellular components, and (C) molecular function.

To investigate the regulation of POI-related target genes of *miR-146a*C>G and *miR-196a2*T>C, we used microarray data. To analyze the function of those genes, we used DAVID software, which queries for biological activities according to GO annotations. The DAVID functional analysis of *miR-146a*C>G (Fig 2A) showed that the main biological processes altered between the wild-type C allele and the variant types were cell differentiation (22 and 4 genes, respectively), metabolism (30 and 17 genes, respectively), cell growth (22 and 4 genes, respectively), and regulation of genes often involved in reproductive diseases 28 and 11 genes, respectively). The cellular components altered between wild-type and variant-type *miR-146a*C>G were intracellular (79 and 34genes, respectively) and extracellular (21 and 3 genes, respectively) (Fig 2B). The main molecular functions of *miR-146a*C>G altered between wild type and variant type were nucleic acid binding (61 and 24 genes, respectively), calcium ion binding (12 and 2 genes, respectively), and protein binding (9 and 3genes, respectively) (Fig 2C). The DAVID functional analysis for *miR-196a2*T>C (Fig 2A) showed that the main biological processes altered between the wild-type T allele and variant type were regulation of gene expression (23 and 55 genes, respectively), metabolism (35 and 70 genes, respectively), and cell organization and biogenesis (16 and 31 genes, respectively). The main cellular components altered between wild-type and variant-type *miR-146a*C>G were intracellular (76 and 145genes, respectively) and extracellular (12 and 18genes, respectively) (Fig 2B). The main molecular function of *miR-146a*C>G altered between wild type and variant type were nucleic acid binding (62 and 125genes, respectively), calcium ion binding (8 and 13genes, respectively), transferase (19 and 30genes, respectively), and transcription factors (13 and 34genes, respectively)(Fig 2C).

### Validation of microarray data and POI-related *miR-146a* target gene identification

To examine the reliability of the array data, we randomly selected eight mRNAs detected by microarray (S1 Fig) to confirm their expression in a granulosa cell line using qPCR. The results from the qRT-PCRs were consistent with the microarray data (S1 Fig). Using the TargetRank software (http://genes.mit.edu/targetrank/), we found that the alternative mature forms of *miR-146a*-3p and *miR-196a2*-3p were predicted to have several hundred target genes (data not shown). Using a Target Rank score of ≥0.35, 214 and 166 genes were found for *miR-146a*-3pC and for *miR-146a*-3pG, respectively (S1 Table).

**Fig 3:**
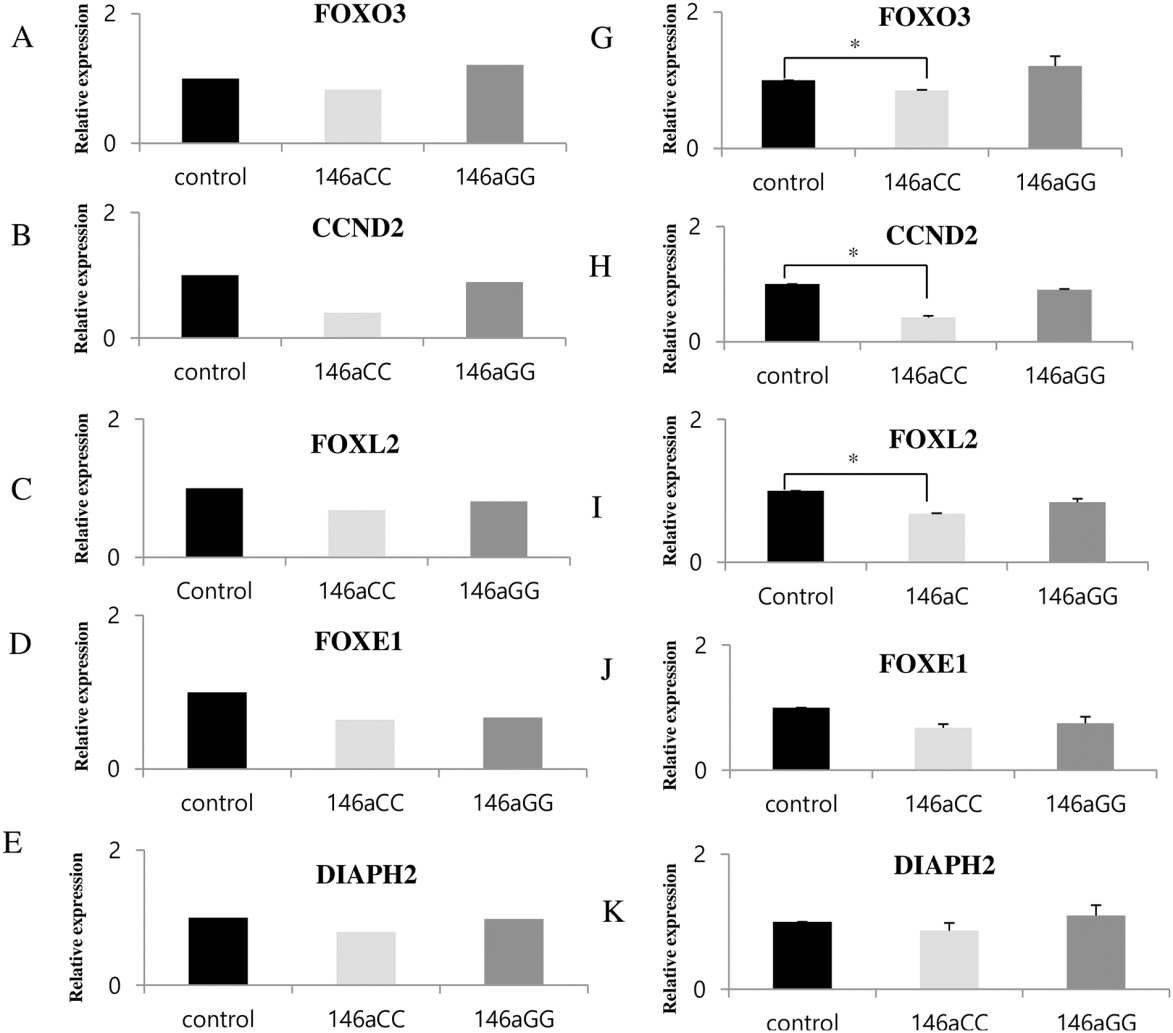
Expression of POI-related *miR-146a*C>G target genes. Validation of the expression of down-regulated genes from microarrays (A-F) and qPCR-arrays (G-L). Transient transfection of pGL4.13 plasmids with FOXO3, FOXl2, ESR1, BBS9, DIAPH2, and FOXE1 in the KGN cell line. mRNA expression levels were normalized to those of GAPDH expression. The data indicate the mean values with the standard deviation relative to miR-mimic negative expression from three independent experiments. Control (miR-scrambled + pGL4.13–3’UTR of each target gene). *P<0.05 was considered statistically significant.

Because each mature miRNA bound to a distinct set of target genes, different target genes were affected by the miRNAs produced by G or C homozygotes (*miR-146a* and *miR-146a*-3pC or *miR-146a*-3pG, respectively). We chose POI-related target genes from micro array data (*FOXO3*, *FOXL2*, *DIAPH2*, *BDNF*, *CCND2*, and *FOXE1*) (Table 1, S2 Table). No distinct set of POI-related target genes produced by *miR-196a2*-3p T or C homozygotes were found. To examine the reliability of the POI-related target gene expression, we selected six mRNAs detected by microarray (Fig 3A–3F). Six individual target mRNAs were further examined in an independent set of RNA samples using the qPCR-array and Real time PCR. As shown in Fig 3, individual expression using the micro array confirmed the result of the Real time PCR (Fig 3A–3F) and qPCR-array (Fig 3G–3L) analysis for all six genes.

### Regulation of 3’-UTR of target genes from the *miR-146a* polymorphisms

**Fig 4:**
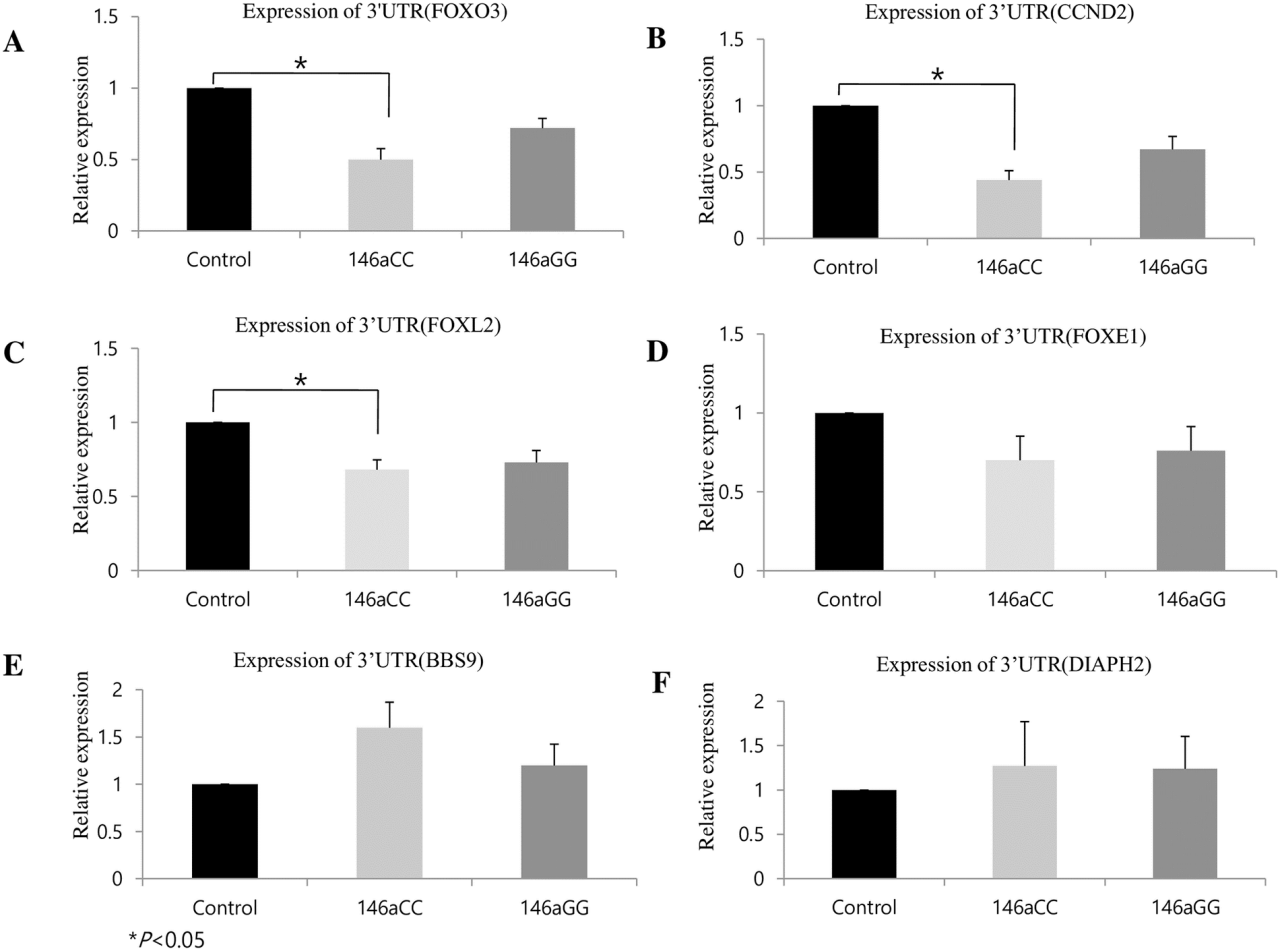
Validation of POI-related *miR-146aC>G* target genes. Validation of the expression using qRT-PCR. Transient transfection of pGL4.13 plasmids with FOXO3, FOXl2, ESR1, BBS9, DIAPH2, and FOXE1 in the KGN cell line. mRNA expression levels were normalized to those of GAPDH expression. The data indicate the mean values with the standard deviation relative to miR-mimic negative expression from three independent experiments. Control (miR-scrambled + pGL4.13–3’UTR of target gene). *P<0.05 was considered statistically significant.

To analyze the effect of *miR-146a*C>G on mRNA levels of six putative target genes (*FOXO3*, *FOXL2*, *DIAPH2*, *BDNF*, *CCND2*, and *FOXE1*) identified from microarray and qPCR-array data, we constructed an expression vector system and quantified the abundance of candidate mRNAs reported to be regulated by *miR-146a* using real-time PCR (Applied Biosystems, Foster City, CA, USA)(Fig 4). We observed a significant reduction in mRNA expression in the cells co-transfected with both pcDNA3.1-*miR-146a*C and pGL-4.13–3’UTR(FOXO3), pGL4.13–3’UTR(CCND2), or pGL4.13–3’UTR(FOXL2) compared to the cells co-transfected with both pcDNA3.1-*miR-146a*G and pGL4.13–3’UTR(FOXO3), pGL4.13–3’UTR(CCND2), or pGL4.13–3’UTR(FOXL2), indicating *miR-146a*C>G could regulate the 3’UTRs of *FOXO3*, *CCND2*, and *FOXL2* mRNAs in the Human granulosa cell line (Fig 4).

**Fig 5:**
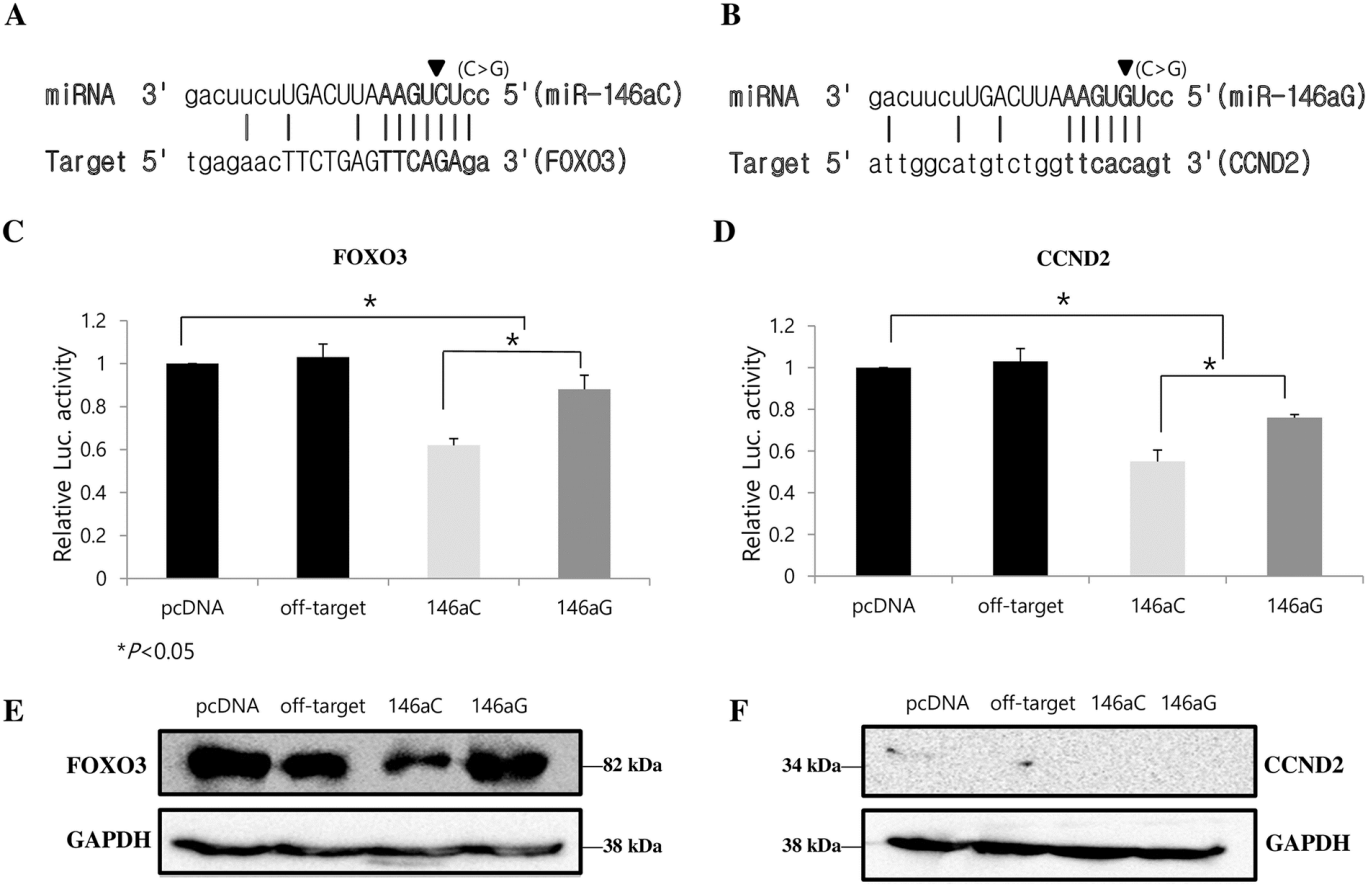
Effects of *miR-146a*C>G on the luciferase reporter gene with the 3’-UTR of FOXO3 and CCND2. Schematic representation of target genes 3´-UTRs that have presumed *miR-146a*-3p-C and *miR-146a*-3p-G binding sites in conserved regions (A and B). Transient transfection of pGL4.13 plasmids with FOXO3 (C) and CCND2 (D) into the KGN cell line with *miR-146aC>G* in pcDNA3.1. Luciferase expression levels were normalized using renilla luciferase expression. Western blot was used to detect FOXO3 and CCND2 protein levels in each group (E and F). KGN cells were transfected with *miR-146a*C or *miR-146a*G in pcDNA3.1 or controls. Protein expression levels were normalized using GAPDH expression. The expression data are the mean ± standard deviation of three independent experiments normalized to the miR-mimic (off-target) negative control. Control: miR-scrambled + target construct + pGL4.75, 146aC: *miR-146a*C + target construct + pGL4.75, 146aG: *miR-146a*G + target construct + pGL4.75. *P*<0.05 was considered statistically significant.

To experimentally confirm the interaction between *miR-146a*C>G and *FOXO3* and *CCND2*, we applied reporter gene assays. We cloned fragments of 3’UTR segments of *FOXO3* or *CCND2* into the pGL4.13 expression reporter vector (Promega, Madison, WI, USA). Then, we transfected pGL-4.13–3’-UTR (*FOXO3*) and pGL-4.13–3’UTR (*CCND2*) into KGN cell lines with or without pcDNA3.1-*miR-146a*. Either C or G alleles of *miR-146a* were used in the analyses. After 24 hours, we observed a significant reduction of luciferase activity in the cells transfected with both pcDNA3.1-*miR-146a* and pGL-4.13–3’UTR(*FOXO3*) or pGL-4.13–3’UTR(*CCND2*) compared to the cells transfected with pGL-4.13–3’UTR(*FOXO3*) or pGL-4.13–3’UTR(*CCND2*) alone or with scrambled miRNA (Fig 5C and 5D), indicating *miR-146a*C>G could bind to the 3’UTRs of *FOXO3* and *CCND2* mRNAs *in vitro*.

### Endogenous regulation of target proteins from the *miR-146a* polymorphisms

Endogenous FOXO3, CCND2, FOXE1 and FOXL2 expression levels were detected by performing Western blots of KGN cells transfected with miR-146aC, miR-146aG or control (pcDNA3.1 and a miRNA-mimic off-target control) (Fig 5E and 5F and S2 Fig). FOXO3 protein level was significantly diminished when cells were transfected with *miR-146a*C compared with *miR-146a*G or off-target control, By contrast, CCND2 protein level showed no marked difference when cells were transfected with *miR-146a*C compared with *miR-146a*G or off-target control (Fig 5E and 5F).

### Clinical characteristics

FSH, LH, and E2 levels of POI patients and controls are summarized in Table 2. Patients with POI had significantly increased FSH and LH levels (p<0.0001, p<0.0001) and Patients with POI had decreased E2 levels compared with control women (p<0.0001). However, hormonal levels were not significantly different between POI patients and control subjects with *miR-146a*C>G and *miR-196a2*T>C (Table 3).

**Table 2:** Clinical variables of Korean participants in the primary ovarian insufficiency (POI) patients and control groups.

Characteristics	Controls	POI patients	*P* value
Age mean ± SD (range)	33.34 ± 5.70 (23–43)	31.34 ± 4.97 (21–43)	NS
FSH (mIU/ml) mean ± SD	8.12 ± 2.85	66.46 ± 14.11	<0.0001
LH (mIU/ml) mean ± SD	3.32 ± 1.761	26.23 ± 10.68	<0.0001
Estradiol (pg/ml) mean ± SD	26 ± 14.75	7.93 ± 2.59	<0.0001

Abbreviations: SD, standard deviation; NS, not significant; FSH, follicle-stimulating hormone; LH, luteinizing hormone; *P* value by t test.

**Table 3:** FSH, LH and E2 levels of control and POI participants based on miRNA polymorphic genotype.

	FSH(mIU/mL) mean ± SD		LH(mIU/mL) mean ± SD		E2(pg/mL) mean ± SD	
Genotypes	control	patients	control	patients	control	patients
*miR-146a*C>G						
CC	8.46 ± 3.53	61.29 ± 13.52	3.3 ± 1.75	26.01 ± 7.19	25.75 ± 12.34	7.91 ± 2.97
CG	8.05 ± 2.44	62.05 ± 14.44	3.44 ± 1.84	26.70 ± 13.98	26.95 ± 16.08	7.62 ± 1.73
GG	7.45 ± 2.39	65.44 ± 14.78	2.88 ± 1.23	25.26 ± 3.78	22.6 ± 15.34	8.93 ± 3.63
*P*	0.511	0.533	0.567	0.856	0.61	0.144
*miR-196a2*T>C						
TT	7.8 ± 2.17	62.09 ± 13.38	2.67 ± 1.22	28.80 ± 17.71	23.52 ± 10.63	8.50 ± 3.40
TC	8.57 ± 3.16	63.58 ± 15.63	3.40 ± 1.99	25.85 ± 9.05	27.60 ± 15.61	7.74 ± 2.24
CC	7.23 ± 2.20	60.30 ± 12.01	3.51 ± 1.26	25.12 ± 5.77	23.70 ± 14.70	7.82 ± 2.51
*P*	0.101	0.501	0.239	0.343	0.383	0.412

Abbreviations: SD, standard deviation; FSH, follicle-stimulating hormone; LH, luteinizing hormone; POI, Primary ovarian insufficiency; *P* value by ANOVA test.

## Discussion

Increasing evidence supports the role of microRNAs in reproductive disorders [24–27]. In this context, evidence supporting the role of *miR-146a* and miR-*196a2* in oocyte maturation and preimplantation embryo development [13, 28] has also been accumulating, which led us to investigate whether these miRNA SNPs (*miR-146a*C>G and *miR-196a2*T>C) influence the risk of POI-related genes in Korean woman.

We previously showed that the combined genotypes and haplotypes of *miR-146a*C>G, *miR-196a2*T>C could be associated with POI in Korean women [12]. We also found that the expression of pre, mature, and mature-3p *miR-146a* with the G allele was significantly lower (*P<*0.05) than that of *miR-146a* with the C allele (Fig 1C), [19]. These results suggest that the polymorphisms in *miR-146a* could change their target gene expression [19]. The recent identification of miRNAs as an important posttranscriptional gene regulator elucidated the role of posttranscriptional gene regulation in reproductive disease [24,25]. In addition, miRNAs are involved in crucial cell processes, such as apoptosis, differentiation, and oncogenesis by regulating signal transduction pathways [29]. To investigate regulation of the POI-related target genes of *miR-146a*C>G and *miR-196a2*T>C, we used microarray data and prediction tools. To analyze the function of those genes from the microarray data, we used DAVID software [30], which provides a comprehensive set of functional annotation tools for investigators to understand biological meaning behind large list of genes according to GO annotations [30]. The functional analysis of *miR-146a*C>G and *miR-196a2*T>C showed that the main biological processes altered between wild type and variant type were cell differentiation, metabolism, cell growth, and regulation of genes often involved in reproductive diseases (Fig 2).

Using TargetRank (http://genes.mit.edu/targetrank/), we found that the alternative mature forms of *miR-146a*-3p and *miR-196a2*-3p were predicted to have several hundred target genes (using a Target Rank score of ≥0.35, 214 and 166 genes were found for *miR-146a*-3pC and *miR-146a*-3pG, respectively, and 55 genes were found for *miR-196a2*-3p T or C; data not shown). Of those genes, we selected the POI-related *miR-146a*C>G target genes *FOXO3*, *FOXL2*, *DIAPH2*, *BDNF*, *CCND2*, and *FOXE1*. Previously, our work suggested that the gene—gene interaction between *miR-146a* and *miR-196a2*could be involved in POI development [12]; however, we did not observe any expression changes associated with *miR-196a2* polymorphisms (Fig 1C), [19].

To further determine the function of the differentially expressed target genes of *miR-146a*-C>G, we investigated transcriptional regulation of *FOXO3*, *FOXL2*, *DIAPH2*, *BBS9*, *CCND2*, and *FOXE1* as target genes of *miR-146a*C>G polymorphisms. We found that *miR-146a* leads to a significant regulation of the mRNA levels of *FOXO3*, *FOXL2*, *and CCND2* compared to controls (Fig 4). Of note, *FOXO3* is targeted by *miR-146a*-3pC. Because Foxo3 functions as a master regulator and potent suppressor of primordial follicle activation, loss of function in the mouse leads to POI due to global follicle activation [31]. The *FOXO3* gene encodes the forkhead family of transcription factors, which is characterized by a distinct forkhead domain [32]. *FOXO3* likely functions as an apoptosis trigger by activating genes necessary for cell death [33]. Numerous studies report that *FOXO3*-deficient ovaries exhibit defects in oocyte apoptosis [34–36]. In addition, mutations in FOXO1 and FOXO3 have been identified in women with POI [37].

We also found that *CCND2* is regulated by *miR-146a*C>G. *CCND2* involved in Wnt signaling, cell-cycle pathways, and adhesion molecule formation [38]. In addition, Wnt signaling and cell-cycle pathways are crucial in POI patients [39]. *CCND2* plays an important role in initiating the early-to-mid G1 phase transition and is required for granulosa cell proliferation during ovarian folliculogenesis [40]. These results raise the possibility that miRNA polymorphisms may contribute to dysregulation and/or functional variations of the CCND2, FOXO3. Because *FOXL2* is suggested to play a role in ovarian development and function [41], it may be an important mediator of ovarian development through *miR-146a*. However, it remains unknown whether *FOXL2* is a target of *miR-146a* activity.

To experimentally confirm the interaction between *miR-146a*C>G and FOXO3 or CCND2, we used reporter gene and Western blot assays. We observed a marked luciferase level reduction in cells co-expressing *miR-146a*C and the 3’-UTR of FOXO3-luc. or the 3’-UTR of CCND2-luc. However, luciferase levels were not reduced as efficiently in cells co-expressing miR-146aG and the 3’-UTR of FOXO3-luc. or the 3’-UTR of CCND2-luc. (Fig 5C and 5D).

These findings suggest that *miR-146a* polymorphisms differentially affect *CCND2* and *FOXO3* expression. We observed that *miR-146a* directly binds the 3’UTR of the target gene potentially regulating the expression of target gene mRNA. More interestingly, we observed that the binding capacity of *miR-146a* to the 3’-UTR of target genes was significantly stronger in cell lines transfected with the wild-type C allele compared with those transfected with the G allele. This observation indicates that the binding capacity was significantly different between common and variant alleles (C and G type). We also confirmed that FOXO3 was decreased by western blot (Fig 5E). These results are consistent with our observation that the wild-type C allele had higher levels of mature *miR-146a* than the mutant G allele.

A recent study showed that *miR-146a*, *miR-27a*, *miR-23a*, and *miR-126* were highly expressed in plasma from POI patients compared with controls; fold change was 5.19, 2.98, 2.75, and 2.29 respectively [42]. Furthermore, in isolated ovarian granulosa cells from patients with POI, the expression of *miR-146a* was markedly increased [43]. These results suggest that *miR-146a* is significantly upregulated in POI patients compared with normal controls. In addition, numerous studies have implicated *miR-146a* in cell apoptosis [44–46]. *miR-146a* has been reported to contribute to granulosa cell apoptosis [47]. These results indicate that *miR-146a* plays an important role in the apoptosis of ovarian granulosa cells in patients with POI. Our previous association data also indicated that the *miR-146a*C allele in combination with the *miR-196a2*T allele increased POI risk. By contrast, the *miR-146a*G allele in combination with the *miR-196a2*T allele reduced POI risk. (OR: 0.193, 95% CI: 0.037–1.002, *P*<0.05) [12]. Thus, the C-allele type of *miR-146a* interacts more effectively with target genes (*FOXO3 and CCND2*) than the G-allele type, increasing the negative effects of POI. A previous report showed that targeted disruption of FOXO3 in granulosa cells leads to the production of ovarian-derived factor(s) that potently suppresses pituitary FSH biosynthesis [48]. Therefore, we examined if polymorphisms of *miR-146a*C>G influence FSH, LH and E2 levels via regulation of FOXO3. We used ANOVA to determine if there are any statistically significant differences between hormonal levels (FSH, LH, E2) and *miR-146a*C>G. While no significant difference was found, there was a trend of increasing FSH levels with the *miR-146a*G polymorphic variant.

Taken together, our data suggest a model where *miR-146a* might play a role in the regulation of *FOXO3* and *CCND2*, and a functional genetic variant in *miR-146a* can alter the expression of mature *miR-146* and thereby affect POI-related target gene expression. Therefore, it is biologically plausible that the *miR-146a*C>G genetic variant may influence POI susceptibility by altering the expression of *miR-146a* target genes (e.g., *FOXO3*, *FOXL2* and *CCND2*).

In this work, the miRNA target sites of interest in the 3’-UTR of (*FOXO3* and *CCND2*) were cloned downstream of a reporter gene (luciferase). Transfection of wild-type and variants of *miR-146a* resulted in altered expression of luciferase and FOXO3 proteins, indicating that polymorphisms of *miR-146a*C>G could be responsible for the differential translation in this in vitro system. However, the 3′-UTRs may contain multiple targeting sequences and other regulatory elements. Further assays to test each miRNA targeting sequence are needed. One limitation of our study is that we could not compare expression levels of miRNAs and target genes in ovary samples collected from patients with POI who had been miRNA genotyped because we had limited access to these samples. While we have not identified the underlying mechanisms by which *miR-146a*C>G polymorphisms affect the development of POI, the data suggest that *miR-146a*C>G polymorphisms could contribute to regulation of POI-related target genes, specifically FOXO3. Further studies of other pre-miRNA polymorphisms in diverse ethnic populations will advance our understanding of the role of miRNA polymorphisms in POI and POI target gene regulation.

## Conclusion

Several genetic studies using the candidate gene approach have now been undertaken, and many potentially causal gene variants have been identified in POI [4, 49,50]. However, there are no examples that show how the SNP polymorphisms at the miRNA level affect the 3’-UTR of miRNA target genes in POI. We found evidence that the genetic polymorphism in *miR-146a*C>G regulated several POI-related target genes. Previous reports showed that polymorphisms in the *miR-146a* precursor (rs2910164) altered the expression of pre- and mature *miR-146a* [51–53]. To the best of our knowledge, this is the first report to evaluate POI-related gene regulation by *miR-146a* polymorphisms. As more experimental information about transcriptional regulation of miRNA genes themselves and their target genes is reported, we could extend the current study to have a deeper understanding of miRNA target gene regulation and the relationship between the regulation of miRNA genes and that of their targets in POI.

## Supporting information

S1 Fig Validation of microarray data by real-time PCR. mRNA was extracted following *miR-146a*C and *miR-146a*G expression. The expression of mRNAs relative to GAPDH RNA was determined by real-time PCR.
(TIF)

S2 Fig Western blot was used to detect FOXE1 and FOXL2 protein levels in each group. KGN cells were transfected with *miR-146a*C or *miR-146a*G in pcDNA3.1 or controls. Protein expression levels were normalized using GAPDH expression.
(TIF)

S1 Table Target genes of *miR-146a*C>G predicted using TargetRank software. (DOCX)

S2 Table Putative miR-*146a* binding target genes. (DOCX)
