# Supplementary figures and images for: Single nucleotide polymorphisms at miR-146a/196a2 and their primary ovarian insufficiency-related target gene regulation in granulosa cells
Source: PLoS One. 2017 Aug 25;12(8):e0183479. doi: 10.1371/journal.pone.0183479 (PMC5571913; doi:10.1371/journal.pone.0183479)

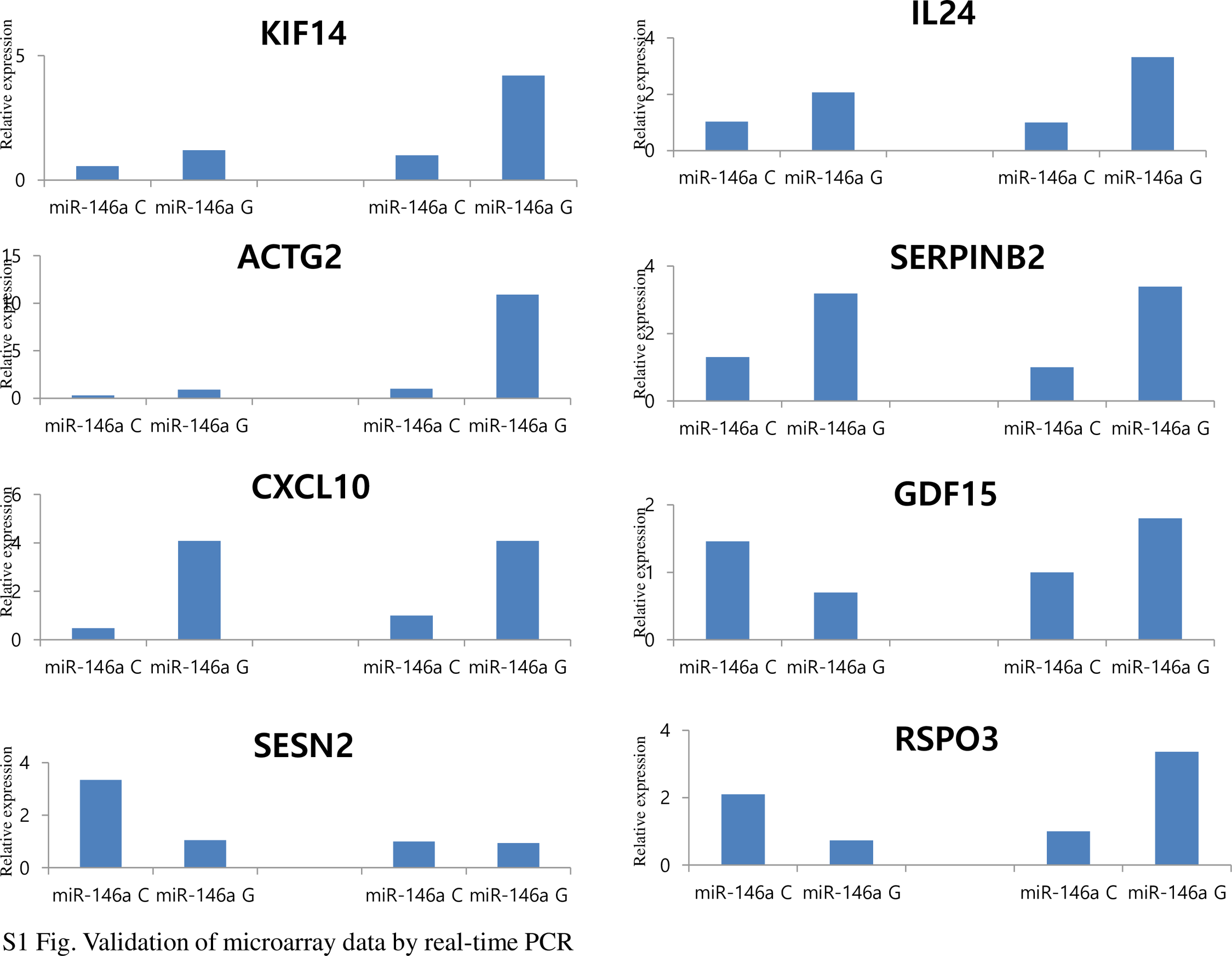

Supplement: S1 Fig — mRNA was extracted following miR-146aC and miR-146aG expression. The expression of mRNAs relative to GAPDH RNA was determined by real-time PCR. (TIF) [file pone.0183479.s001.tif]

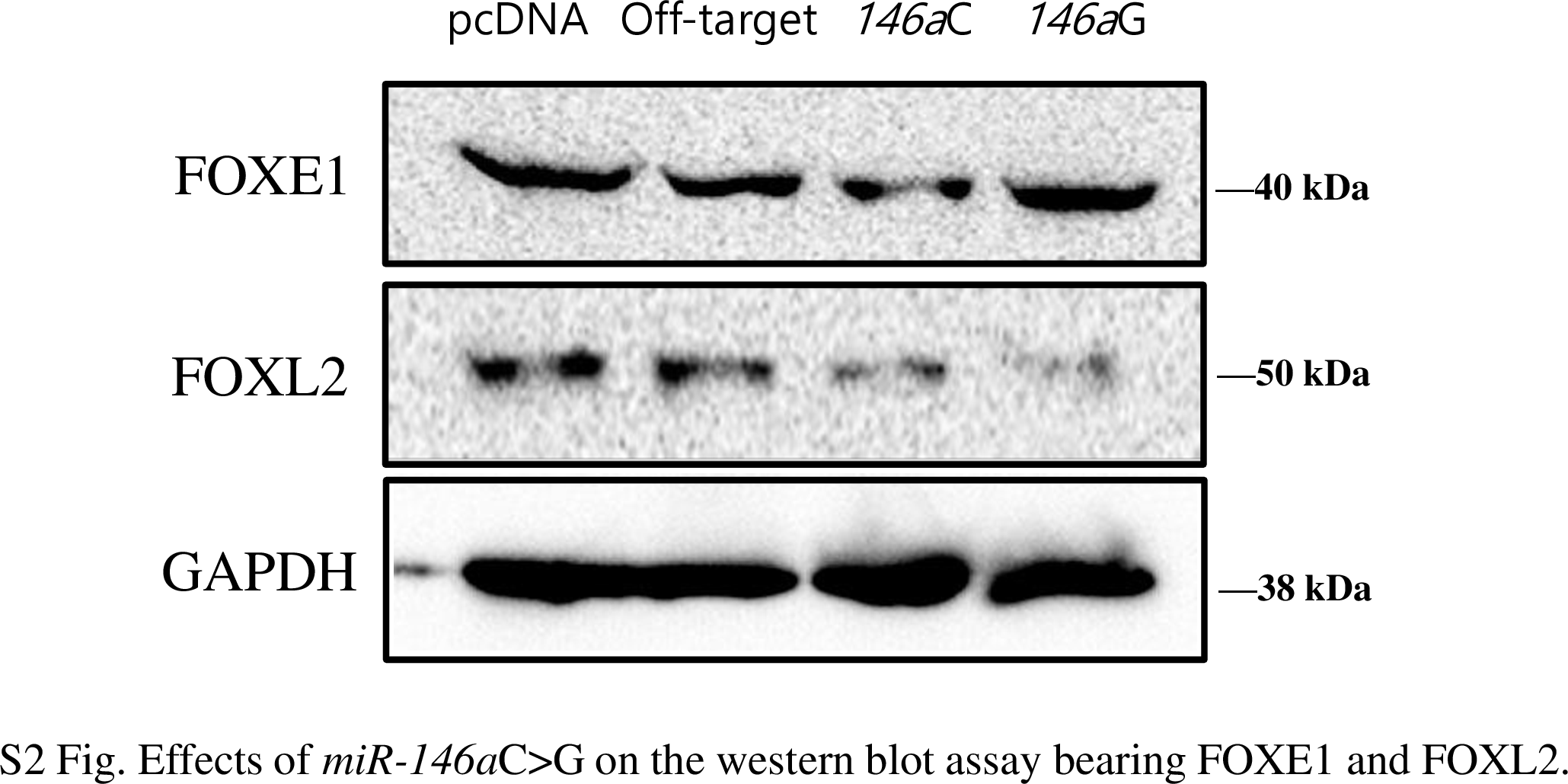

Supplement: S2 Fig — KGN cells were transfected with miR-146aC or miR-146aG in pcDNA3.1 or controls. Protein expression levels were normalized using GAPDH expression. (TIF) [file pone.0183479.s002.tif]
