# Supplementary material for: Single nucleotide polymorphisms at miR-146a/196a2 and their primary ovarian insufficiency-related target gene regulation in granulosa cells
Source: PLoS One. 2017 Aug 25;12(8):e0183479. doi: 10.1371/journal.pone.0183479 (PMC5571913; doi:10.1371/journal.pone.0183479)
Supplement: S1 Table — (DOCX) [file pone.0183479.s003.docx]

| **S1 Table. Target genes of *miR-146a*C>G predicted using TargetRank software*** | | |  |  |
| --- | --- | --- | --- | --- |
| Gene name | Gene isoform discription | RefseqID | Score | Genotype |
| DIAPH2 | diaphanous 2 isoform 156 | NM_006729 | 0.435 | *miR-146a*G |
| BBS9 | parathyroid hormone-responsive B1 isoform 2 | NM_198428 | 0.506 | *miR-146a*G |
| CCND2 | cyclin D2 | NM_001759 | 0.544 | *miR-146a*G |
| FOXE1 | forkhead box E1 | NM_004473 | 0.209 | *miR-146a*G |
| FOXO3 | forkhead box O3A | NM_201559 | 0.409 | *miR-146a*C |
| *targetRank software (http://genes.mit.edu/targetrank/) | |  |  |  |
