# Supplementary material for: Single nucleotide polymorphisms at miR-146a/196a2 and their primary ovarian insufficiency-related target gene regulation in granulosa cells
Source: PLoS One. 2017 Aug 25;12(8):e0183479. doi: 10.1371/journal.pone.0183479 (PMC5571913; doi:10.1371/journal.pone.0183479)
Supplement: S2 Table — (DOCX) [file pone.0183479.s004.docx]

| **S2 Table.** **Putative *miR-146a* binding target genes** | | |  |
| --- | --- | --- | --- |
| target gene | Prediction* | Micro-array expression‡ | References** |
| FOXO3 | *miR-146a*C | 0.83±0.02 | Vinci et al. |
| target gene | *miR-146a*G | Micro-array expression | POI-related |
| CCND2 | *miR-146a*G | 0.51±0.04 | Zhang et al. |
| DIAPH2 | *miR-146a*G | 0.85±0.03 | Bione et al. |
| BBS9 | *miR-146a*G | 0.81±0.07 | Kang et al. |
| FOXE1 | *miR-146a*G | 0.77±0.09 | Watkins et al. |
| FOXL2 | *miR-146a*G | 0.56±0.07 | Harris et al. |
| *miR-146aC>G target prediction: targetRank software (<http://genes.mit.edu/targetrank/>)  ‡Mean fold change±SD (t-test p-value less than 0.05 in 146a-C and 146a-G) | | | |

**Target genes known to be involved in POI.

References:

Vinci G, Christin-Maitre S, Pasquier M, Bouchard P, Fellous M & Veitia RA 2007 FOXO3a variants in patients with premature ovarian failure. Clinical Endocrinology 68 495–497.

Zhang Q, Sun H, Jiang Y, Ding L, Wu S, Fang T, Yan G, Hu Y. MicroRNA-181a suppresses mouse granulosa cell proliferation by targeting activin receptor IIA. PLoSOne. 2013; 8(3):e59667.

Bione S, Sala C, Manzini C, Arrigo G, Zuffardi O, Banfi S, Borsani G, Jonveaux P, Philippe C, Zuccotti M, Ballabio A, Toniolo D. A human homologue of the Drosophila melanogaster diaphanous gene is disrupted in a patient with premature ovarian failure: evidence for conserved function in oogenesis and implications for human sterility. Am J Hum Genet. 1998 Mar;62(3):533-41.

HyunJun Kang, Seung Ku Lee, Min-Ho Kim, JiHyun Song, Su Jin Bae, Nam Keun Kim, Sook-Hwan Lee, KyuBum Kwack. Parathyroid hormone-responsive B1 gene is associated with premature ovarian failure. Hum Reprod (2008) 23 (6): 1457-1465.

Watkins WJ, Harris SE, Craven MJ, Vincent AL, Winship IM, Gersak K, Shelling AN. An investigation into FOXE1 polyalanine tract length in premature ovarian failure. Mol Hum Reprod. 2006 Mar;12(3):145-9.

Harris SE, Chand AL, Winship IM, Gersak K, Aittomäki K, Shelling AN. Identification of novel mutations in FOXL2 associated with premature ovarian failure. Mol Hum Reprod. 2002 Aug;8(8):729-33.
